# Occurrence and Characterization of *Salmonella* Isolated from Table Egg Layer Farming Environments in Western Australia and Insights into Biosecurity and Egg Handling Practices

**DOI:** 10.3390/pathogens9010056

**Published:** 2020-01-13

**Authors:** Hamid Reza Sodagari, Ihab Habib, Scott Whiddon, Penghao Wang, Arkan Baraa Mohammed, Ian Robertson, Stan Goodchild

**Affiliations:** 1 School of Veterinary Medicine, College of Science, Health, Education and Engineering, Murdoch University, Perth 6150, Australia; Hamidreza.Sodagari@murdoch.edu.au (H.R.S.); P.Wang@murdoch.edu.au (P.W.); dr.arkanmohammed@gmail.com (A.B.M.); I.Robertson@murdoch.edu.au (I.R.); 2 Veterinary Medicine Department, College of Food and Agriculture, United Arab Emirates University (UAEU), Al Ain P.O. Box 1555, UAE; 3 Department of Health Western Australia, 189 Royal Street, East Perth District, Perth 6004, Australia; Scott.Whiddon@health.wa.gov.au (S.W.); Stan.Goodchild@health.wa.gov.au (S.G.)

**Keywords:** *Salmonella*, eggs, Western Australia, layers, biosecurity

## Abstract

The aim of this study was to investigate the occurrence and distribution of *Salmonella* in commercial layer farming environments of 26 flocks belonging to seven egg businesses (free-range and barn-laid) in Western Australia (WA). Between November 2017 and June 2018, a total of 265 environmental samples of dust, feed, water, pooled feces, and boot swabs were tested for detection of *Salmonella* according to standard culture-based methods. Isolates were assayed for serovar and subtyped by multilocus sequence typing (MLST). *Salmonella* spp. were recovered from 35% (93/265) of all tested samples. Dust (53.8%, 28/52) and pooled fecal (54.5%, 18/33) samples provided the highest *Salmonella* recovery rates. Nine different *Salmonella* serovars were characterized across the positive (*n* = 93) environmental samples, of which *S.* Typhimurium (60/93, 64.5%) and *S.* Infantis (21/93, 22.5%) were the most prevalent. MLST revealed that all *S*. Typhimurium isolates were of sequence type ST-19. Microbiological screening of *Salmonella* was not routinely practiced in any of the surveyed egg businesses. Some of the egg businesses exhibited variable levels of compliance with basic biosecurity measures as well as high-risk egg handling practices. Egg businesses in WA should be encouraged to adopt a voluntary program of environmental sampling and verification testing for *Salmonella*. Such voluntary programs will aid in supporting solutions for the management of this pathogen in the human food chain.

## 1. Introduction

Non-typhoidal *Salmonella* species are among the major foodborne pathogens in Australia [1], with many traced human outbreaks attributed to consumption of eggs and egg-based products [2,3]. *S.* Typhimurium is the key endemic serovar in the Australian egg production industry and is the most frequent serovar isolated from human cases [4]. In recent years, there has been a significant expansion of non-cage (free-range and barn-laid) egg production systems around Australia [5] arising from an increase in consumer demand and concerns regarding caged bird welfare [6]. Nevertheless, some researchers hypothesize that non-cage egg production systems may lead to a higher exposure of layer flocks to the outdoor environment, pests, and wildlife vectors [4,7,8]. The growth of non-cage egg production systems, along with the inherent biosecurity and environmental challenges in such systems, have raised concerns regarding the likelihood of pathogens such as *Salmonella* accessing consumer retail eggs in contaminated farm environments [9]. In Western Australia (WA), the number of human salmonellosis cases has dramatically increased and has been higher than in any other Australian state in recent years [10]. In 2017, the number of cases in WA was more than double the previous five-year average, with eggs and egg-based dishes emerging as the key culprits in several *Salmonella* foodborne outbreaks [10]. At present, there is no published research on *Salmonella* status in table egg layer farm environments in WA, a state that occupies the entire western third of Australia. To understand the epidemiology of salmonellosis events in WA, it is important to generate a baseline status for *Salmonella* contamination in the layer farm environment. The aims of this study were: (1) to establish a baseline survey of the occurrence of *Salmonella* in egg layer farming environments in WA; (2) to characterize identified *Salmonella* isolates based on their serovars and multilocus sequence type (MLST) diversity; and (3) to collect industry profile information on common primary production practices and identify areas in which egg businesses may require further assistance in meeting biosecurity and compliance requirements.

## 2. Results

### 2.1. The Occurrence of Salmonella in the Layer Farm Environments

*Salmonella* was isolated from the environments of all seven egg businesses and from 23 of the 26 (88.5%) sampled flocks. *Salmonella* was recovered from all (11/11) of the free-range flocks as well as from 80% (12/15) of the barn-laid flocks (Table 1).

Of 265 tested environmental samples, 93 (35.0%) were positive for *Salmonella.* Pooled fecal samples showed the highest rate of *Salmonella* (54.5%, 18/33) recovery, followed by dust samples (53.8%, 28/52). Further, *Salmonella* recovery from inside-shed boot swabs (42.5%, 17/40) was comparable to the recovery rate from outside-shed boot swabs (40.9%, 9/22). However, feed samples collected from inside the sheds showed slightly higher rates of *Salmonella* recovery (30.4%, 14/46) than feed samples from silos (26.9%, 7/26,). *Salmonella* was not recovered from any of the tested water samples (*n* = 46) (Table 1).

### 2.2. Diversity of Salmonella Serovars and Sequence Types

In the present study, nine different non-typhoidal serovars were identified from 93 *Salmonella* culture-positive environmental samples. *S.* Typhimurium (64.5%, 60/93) and *S.* Infantis (22.5%, 21/93) were by far the most prevalent serovars, followed by *S.* Muenchen (4.3%, 4/93), *S.* Tennessee (2.1%, 2/93), *S.* Orion (2.1%, 2/93), *S.* Kiambu (1.0%, 1/93), *S.* Alsterdorf subs. II (1.0%, 1/93), *S.* Anatum (1.0%, 1/93), and *S.* Choleraesius v. Decatur (1.0%, 1/93).

Results presented in Table 2 demonstrate that although *S.* Typhimurium was the only detected serovar in the environmental samples collected from egg businesses D and E, there was more than one serovar detected in most of the other egg businesses. *S*. Typhimurium and *S*. Infantis co-existed in flock houses sampled from egg businesses F and G. Moreover, egg businesses A and B harbored four and three different serovar in their flocks, respectively (Table 2). Thirty-three *S.* Typhimurium isolates recovered from different flocks were further subtyped using MLST. All 33 *S.* Typhimurium isolates were characterized as ST-19.

### 2.3. Production Management, Biosecurity, and Egg Handling Practices

A descriptive overview of the questionnaire results obtained from the farm management teams of the seven participating egg businesses is summarized in Table 3. All of the businesses had in-house quality assurance systems, and almost all had adopted a written food safety management statement. A written/documented Hazard Analysis and Critical Control Point (HACCP) system was also present in most of the farms. Nevertheless, few farm managers and staff followed any kind of either formal or in-house training on aspects of HACCP, food safety principles, or biosecurity. The results of the questionnaire also indicated that the egg businesses did not always seek vaccinations against *Salmonella* for their flocks (Table 3). Further, the summary of answers indicated a variable level of compliance with basic biosecurity measures. Areas observed as needing improvement included systematic cleaning and sanitization of sheds between production cycles, standards for disinfection of incoming vehicles to farms, and regular change of workers’ clothes while moving between sheds or instructing farm workers to wear personal protective equipment (Table 3). Other biosecurity gaps included inadequate or absent handwashing facilities and boot sanitization dips in many of the farms, poor maintenance of pest control stations and baits, and inadequate practices to restrict rodent infestations and access to feed and sheds.

Some high-risk egg handling practices were also observed. For instance, three of the seven (42.9%) egg businesses indicated that the storage period prior to grading was as long as 48 h. Moreover, in four of the seven egg businesses (57.1%), pre-graded eggs were typically stored at ambient temperatures (rather than being refrigerated or kept at 15 °C). Finally, the sanitizing frequency of egg handling equipment was variable in terms of frequency and procedure. Daily sanitization of egg handling equipment occurred in four (57.1%) of the visited farms, while weekly (14.3%, 1/7) and even monthly (14.3%, 1/7) sanitization was reported by the remainder.

## 3. Discussion

### 3.1. The First Insight on the Status of Salmonella in Egg Businesses in WA

Microbiological surveillance of farm environments may be beneficial for predicting potential *Salmonella* introduction and colonization of layer flocks and subsequent contamination of table eggs [11]. This is the first study to investigate the occurrence of *Salmonella* in commercial egg businesses in WA. The results of the present study supply new knowledge on the diversity of *Salmonella* serovars and Sequence Types (STs) and provide industry profile information on common primary production practices in WA egg businesses.

All of the visited egg businesses in the present study in WA were positive for *Salmonella* (100%, 7/7). Compared with our results, farm-level surveys conducted in the Australian states of Queensland (Qld) [12] and New South Wales (NSW) [13] found a lower occurrence of *Salmonella* (33.3% and 47.0%, respectively). Similarly, lower rates were also found in countries such as New Zealand (20%, 4/20) [14], Korea (59.3%, 19/32) [15], and Argentina (43.3%, 13/30) [16]. Additionally, our results indicate that *S.* Typhimurium was present in five of the seven (71.5%) surveyed farms, which is considerably higher compared with findings from the Australian states of Qld and NSW, which reported detection of environmental *S.* Typhimurium in 13.5% and 20% of surveyed farms, respectively [12,13]. Our results were also higher than those of a New Zealand survey of *S.* Typhimurium in the environment of layer farms (16.6%, 3/18) [14]. The higher rates of *Salmonella*, particularly *S.* Typhimurium, detected in the present study compared with those found in other parts of Australia and New Zealand may be attributable to the climatic conditions in different geographical areas, as well as to *Salmonella* infection status of birds and the effectiveness of cleaning and other hygiene and biosecurity measures [17]. *S.* Typhimurium has been the dominant serovar in recent human salmonellosis cases in Australia and has been frequently implicated in causing egg-borne human salmonellosis outbreaks, which is an increasing public health concern [18,19]. In WA, the reported number of human salmonellosis cases has increased dramatically compared with other Australian states, and *S.* Typhimurium was the major species responsible for most of the egg-linked outbreaks in WA [10].

According to our results (Table 1), *Salmonella* was recovered from all of the free-range egg businesses flocks and 80% of the barn-laid egg businesses flocks. These results contrast with investigations conducted in Qld (free-range [30%, 3/10] and barn-laid [50%, 1/2]) [12] and NSW (free-range [40%, 12/30] and barn-laid [100%, 4/4]) [13]. There is conflicting evidence on the effect of housing as a risk factor for *Salmonella* shedding. A previous study in Belgium suggested that there is no consistent conclusion on the interaction of stress with different housing systems [20]. In contrast, a study in the United States found that free-range hens are exposed to a higher number of environmental stressors, including weather variations, heat stress, and predation, as well as contact with wild birds, which leads to a high chance of *Salmonella* shedding in environment [8]. However, more studies are required to understand the epidemiology of *S.* Typhimurium in free-range egg businesses; especially that such production system has seen growth driven by consumer demands, focus on animal welfare, as well as the environmentally friendly production of eggs in Australia and other countries [9]. 

### 3.2. Salmonella Recovery from Environmental Samples

According to our results, pooled fecal (54.5%, 18/33) and dust (53.8%, 28/52) samples had the highest rate of *Salmonella* recovery. Similar to our findings, investigations in South Australia (SA) also found that dust samples had the highest occurrence of *Salmonella*, with recovery rates ranging from 14.1% (10/70) [9] to 41% (29/70) [4], which are lower than our results. A recent study in New Zealand [14] found that dust samples had the highest rate of *Salmonella* recovery (28.3%, 19/67), which aligns with our findings even though the rates were lower. Compared with our findings, lower rates of *Salmonella* were found in fecal samples originating from layer farm environments in SA (11.7%, 5/40) [9] and NSW (17%, 15/90) [13] as well as in other parts of the world, including New Zealand (10.4%, 7/67) [14], Korea (41.8%, 28/67) [15], and Kosovo (11%, 22/200) [21].

For routine verification and voluntary self-monitoring of egg businesses in WA, fecal and dust samples are highly recommended as the most significant indicators of present and prior *Salmonella* infection in flocks, respectively. Reintroduction of *Salmonella* to flocks has been attributed to residual *Salmonella* contamination on the layer farm [22,23]. It is also hypothesized that *Salmonella* has a greater ability to persist in dust than in other environmental samples [9]. Therefore, dust samples provide an excellent verification of the effectiveness of cleaning and disinfection. In free-range flocks, it is challenging to clean and disinfect ranging areas, however, effective sanitation by removing dust from different surfaces in sheds, especially between production cycles, is shown to be valuable in reducing the level of *Salmonella* contamination [24].

According to our results, the level of *Salmonella* recovered from inside-shed feed samples (30.4%, 14/46) was higher than that found in similar samples in a farm environment survey in NSW (17%, 17/101) [13]. Further, *Salmonella* was also recovered from the tested silo feed samples (26.9%, 7/26) in the present study. Compared with our results, a lower rate of *Salmonella* recovery was reported for stockfeed samples in investigations conducted in Qld (0%, 0/21) [12], NSW (11%, 3/27) [13], and New Zealand (3%, 1/33) [14]. In the present study, *S.* Typhimurium was the serovar detected in 57.1% (4/7) of the stockfeed samples found to be culture-positive for *Salmonella*. However, this serovar was not detected in any of the *Salmonella*-positive stockfeed samples in the NSW investigation [13]. A recent 2019 survey from New Zealand reported that only 1 of 33 tested feed samples was positive for *Salmonella,* which was serotyped as *S.* Thompson [14]. The limited number of local stockfeed supplier companies for egg businesses in WA along with the abundance of *S.* Typhimurium recovery from in-silo feed samples warrants a trace-back verification of the status of *Salmonella* in primary stockfeed production lines. Contaminated feed ingredients such as animal protein sources or the use of contaminated vehicles for distributing feed may play a significant role in the spread of *Salmonella* on farms [21,25]. Protein sources in poultry feed have been frequently reported to be reservoirs of many *Salmonella* serovars [26,27,28].

### 3.3. Diversity of Salmonella Subtypes Recovered from Environmental Samples

According to our findings, *S.* Typhimurium (64.5%, 60/93) and *S.* Infantis (22.5%, 21/93) were the most common isolates from farm environments in WA. Similar to our findings, *S.* Typhimurium (30%, 39/130) and *S.* Infantis (19%, 25/130) were also reported as predominant serovars in a previous study in NSW [13]. Other Australian investigations have also indicated that these two serovars are among the predominant *Salmonella* serovars in Australian egg farms [9,12,29,30,31]. The results of a recent *Salmonella* survey in layer farms in New Zealand revealed that *S.* Infantis was also the most detected serovar, while *S.* Typhimurium was the third most detected [14]. The majority (84%) of reported foodborne *Salmonella* spp. outbreaks in Australia between 2001 and 2016 were attributed to *S.* Typhimurium, while only 1% of these outbreaks were related to *S.* Infantis [32]. Although human cases associated with *S.* Infantis are not as common as those associated with *S.* Typhimurium, *S.* Infantis has emerged as the fourth most common serovar causing human salmonellosis in Europe in 2014 [33]. 

In the present study, *S.* Typhimurium ST-19 was identified as the common multilocus sequence type circulating in layer farms in WA. Numerous common gastroenteritis-causing *S.* Typhimurium isolates have been attributed to ST-19 [34,35,36]. ST-19 was also the most common in *S.* Typhimurium isolates recovered from chicken carcasses in Iran and India [37] as well as in clinical *Salmonella* strains in Brazil [38].

Our results stress the important role of the egg production sector in the epidemiology of *Salmonella* outbreaks in WA; the abundant recovery of *S.* Typhimurium of ST-19 in layers farming environments as well as the predominance of such subtype in human cases calls for prioritization of *Salmonella* management efforts at the primary egg production sector. 

### 3.4. Biosecurity and Production Practices—Points of Attention

Adherence to basic biosecurity measures is crucial for protecting laying hen farms against the introduction of *Salmonella*, as has been reported in several previous investigations [39,40]. Findings from the questionnaire (Table 3) indicate a variable level of biosecurity training for farmers and other staff members in the surveyed egg businesses. It is important to adopt either formal or in-house training on biosecurity and food safety aspects at the level of farms operating in WA. Such training will provide reassuring refreshment on basic principles, and will attain new workers to acquire the best required practices. A Canadian study on poultry farms indicated that biosecurity compliance is closely related to responsibility, work experience, and education [41].

Routine *Salmonella* screening of layer flocks is not mandatory in Australia. However, monitoring for *S.* Enteritidis for commercial egg producers exporting eggs to overseas markets is required in some Australian states, including NSW, Qld, and Victoria [42]. Thus, not surprisingly, *Salmonella* screening was not routine on the visited farms in the present study in WA. Having a regular monitoring/surveillance program for environmental sampling and testing for *Salmonella* will not only help detect and respond to *Salmonella* infection before it spreads, but will also generate knowledge for developing solutions to control this pathogen at the farm level [40].

Various cleaning and sanitization methods were used by business owners in WA. However, most (71.4%, 5/7) did not consider practicing microbiological verification for cleaning and sanitization between successive flocks. The results of a study conducted in five countries across Europe (Belgium, Germany, Greece, Italy, and Switzerland) indicated that 31.8% of farms with *Salmonella*-positive flocks conducted cleaning and disinfection of henhouses, although there was no information about microbiological verification after cleaning [39]. Given that *Salmonella* can survive in empty sheds for several months, especially in organic material such as dust and manure [43], it may transfer between consecutive production cycles. Therefore, ineffective dry-cleaning procedures such as the mechanical removal of organic material (manure, dust, and feed spills) may play a significant role in the persistence of *Salmonella* on farms [39].

According to our questionnaire survey, farmers noted that rodents and birds had access to the vast majority of the visited sites. Open and unsecured shed doors may also provide easy access for unwanted insects, pests, and predators. Similar to our findings, previous investigations in Australia have also demonstrated the role of environmental vectors in the introduction of *S.* Typhimurium, particularly in free-range layer farms [4]. Another study conducted in three Caribbean countries also revealed that 90% of contaminated farms had problems with rodents [44]. High rodent density in layer flocks leads to a higher rate of *Salmonella* shedding and infection [45]. Compared with most other sources of *Salmonella*, rodents have a higher ability to spread this pathogen from one flock to another [46]. Rodents can maintain asymptomatic latent infections and shed *Salmonella* through their fecal pellets, which can infect chickens through the consumption of mouse fecal pellets in feed [47]. Therefore, rodent control programs in poultry premises, including the repairing of holes and other potential access points for rodents into henhouses, the removal of vegetation around sheds, the application of useful baits and bait placement, may help to control *Salmonella* contamination in layer flocks [48].

In the present study, some high-risk egg handling practices were observed at the visited farms in WA. For instance, three of the seven farms indicated that the storage period before grading was as long as 48 h. Our findings also pointed that in four of the seven farms, pre-graded eggs were typically stored at ambient temperatures (rather than at 15 °C, as recommended by the code of Australian Eggs —a producer-owned corporation). According to the Australian Eggs recommendation, eggs should be stored below 15 °C (±3 °C) on farms, during transport and at the retail outlet [49]; apparently such recommendation is not met in most of the visited farms in WA. *Salmonella* can survive on the surface of the eggs for several weeks [50], thus reducing storage temperature can decrease the multiplication and penetration of *Salmonella* during storage at farms. Egg-collecting areas are one of the most important reservoirs for *Salmonella* cross-contamination. The results of a study in Belgium indicated that *S. enteritidis* was common on equipment and surfaces in egg packing areas on farms [51]. Contamination may occur during and after egg collection via contact with workers, surfaces, and equipment [52]. Therefore, adherence to good practices, as those recommended by the code of Australian Eggs [49], is necessary during egg handling, including sorting, candling, grading, and packaging of eggs, to minimize cross-contamination and eggshell surface damage [52].

Further, our results also revealed that farmers did not consider vaccinating their flocks against *Salmonella.* Vaccination is one of a widely accepted means worldwide of preventing or reducing *Salmonella* shedding [53]. Poor adoption of *Salmonella* vaccination in WA egg businesses is not surprising, as the vaccination culture varies between and within countries—for instance, a 2012 study conducted by Sasaki et al. [54] in Japan showed that one-third of the visited layer flocks had been vaccinated against *Salmonella*. In Australia, there is only one approved live attenuated vaccine against *S.* Typhimurium; the long-term efficacy of this vaccine in commercial layer flocks has been evaluated in previous Australian studies but is still questionable [55,56]. However, it has been shown that vaccination is most effective when applied alongside other biosecurity and sanitation procedures [52]. While vaccination is usually effective to one or a low number of serovars, a suitable microbiota could be effective for a broad range of serovars. The first attempt to find such a suitable microbiota for chicks has been reported recently [57].

## 4. Materials and Methods

### 4.1. Recruitment of Egg Businesses

In the present study, we targeted non-cage (free-range and barn-laid) egg businesses because this sector is the most rapidly emerging in the WA table egg industry (promoted by the state government’s plan for a 10-year phase-out of conventional cages for egg-laying hens) [58]. Field work for this study was conducted between November 2017 and June 2018. An invitation package was sent by mail and email to listed egg businesses (*n* = 27) across WA prior to the commencement of the project. The list of businesses was supplied by the WA Department of Health (Food Unit). The aim of the invitation packages was to communicate the objectives of our research to egg businesses and local governments as well as to assure data confidentiality of the participating businesses. In total, seven egg businesses (7/27 (26%)) voluntarily agreed to participate in the present study. The geographical distribution of the surveyed businesses included metropolitan and non-metropolitan regions across WA. The epidemiological sampling unit in the present study was ‘a flock’, defined in the scope of this study as a group (or batch) of laying hens raised in the same shed. The research team sampled 26 flocks from the participating egg businesses. Inclusion criterion was flocks with a production age of >20 weeks (because the laying capacity is less than 50% at younger ages) [12].

### 4.2. Environmental Sampling

A total of 265 environmental samples were collected from 26 flocks belonging to seven egg businesses. The methodology for collection of each sample type is described in Table 4. The samples consisted of dust (*n* = 52), boot swabs from inside sheds (*n* = 40), boot swabs from outside sheds (*n* = 22), feed from inside sheds (*n* = 46), feed from outside sheds (*n* = 26), and water from inside sheds (*n* = 46). Flock sampling methodology adopted in this study was based on guidelines from the European Union (EU) monitoring program [59] and the United Kingdom National Control Program (UK-NCP) (Commission Regulation (EC) no. 1168/2006) for *Salmonella* in layer hens with some modifications. The EU and UK-NCP guided protocols recommend that pooled fecal samples be collected from the environment of cage-laid flocks; however, in the present study, we included pooled fecal samples (*n* = 33) from barn-laid sheds to assess the added value of this sample type in the setting of non-caged flocks.

Disposable sterile gloves, boot swabs, and bags were used during sample collection. All collected samples from the various flock sheds were labelled and aseptically transferred to the Veterinary Public Health Research Laboratory at Murdoch University under chilled conditions. On arrival at the laboratory, samples were refrigerated at 5 °C, and microbiological testing started on the day of sampling.

### 4.3. Isolation, Identification, and Serotyping of Salmonella

Preparation of the collected samples was performed with some modifications to UK-NCP and EU recommendations as described by Carrique-Mas et al. [60]. Briefly, the bag containing fecal sample was weighed and a corresponding volume of buffered peptone water (BPW) (Oxoid UK) was added to maintain a sample to diluent ratio of 1:1 (1 in 1). The feces and BPW were then homogenized in a stomacher for 1 min to enable thorough mixing of the sample with the diluent. Subsequently, 50 mL of this mixture was homogenized for 1 min with 200 mL of BPW. For each boot swab, the sample (pair) was also homogenized for 1 min in a stomacher containing 225 mL of BPW. Further, Whirl-Pak sample bags containing dust and feed samples were weighed separately and filled with a corresponding volume of BPW to maintain a sample to diluent ratio of 1:9 (1 in 10). Finally, each water sample was filtered using Rapid-Flow™ sterile disposable filter units with a nylon membrane (Nalgene) before the nylon membrane was transferred and vortexed with 45 mL of BPW in sterile falcon tubes.

Isolation of *Salmonella* was performed according the 2017 recommendations of the ISO 6579-1 standard [61]. In the pre-enrichment stage, each homogenized sampling unit was incubated at 37 °C for 36 h. Following pre-enrichment, 1 mL was sub-cultured in Muller Kauffmann tetrathionate novobiocin broth (Oxoid UK) and incubated for 48 h at 37 °C, and 0.1 mL was sub-cultured in modified semi-solid Rappaport Vassiliadis (MSRV) (Oxoid UK) and incubated for 48 h at 41.5 °C. MSRV plates were checked after 24 h for a migration zone (turbid, white halo, with a radius larger than 10 mm), and plates with no migration zones were rechecked after an additional 24 h. Following pre-enrichment of both media, streak cultures were conducted on xylose lysine deoxycholate agar and brilliant green agar (Oxoid UK), which were then incubated at 37 °C for 24 h. Suspected colonies (up to five) were selected from both selective media and transferred onto nutrient agar (Oxoid UK) plates. After incubating nutrient agar at 37 °C for 24 h, the purified suspected colonies were identified to species level by matrix-assisted laser desorption ionization–time-of-flight mass spectrometry (MALDI-TOF MS) using the microflex instrument (Bruker Diagnostics, Germany). All confirmed *Salmonella* isolates (up to five isolates per positive sample) were serotyped (Kauffmann-White scheme) at a nationally accredited reference laboratory (PathWest) in Perth, WA. Isolates from confirmed positive samples were stored at −80 °C for further surveys.

### 4.4. MLST of S. Typhimurium Isolates

DNA was extracted from 33 *S.* Typhimurium isolates recovered from positive samples using the Bioline DNA extraction kit (ISOLATE II, Genomic DNA Kit) according to the manufacturer’s instructions. According to the University of Warwick (http://mlst.warwick.ac.uk/mlst/), seven housekeeping genes (*aroC*, *dnaN*, *hemD*, *hisD*, *purE*, *sucA* and *thrA*) were used for the molecular typing of *Salmonella* isolates. Sequence types (STs) were assigned according to the *S. enterica* MLST database (http://mlst.warwick.ac.uk/mlst/dbs/Senterica).

### 4.5. Biosecurity and Production Practices Questionnaire

A comprehensive questionnaire including approximately 120 variables was developed to benchmark the current production practices of surveyed egg businesses in WA. Egg businesses were asked to voluntarily complete the questionnaire which covered several aspects, including information about locality, production system, quality management system, feed and water supply, layer management, aspects of biosecurity management, and egg handling practices. The key variables of the questionnaire are summarized in Table 3. The present study protocol was approved by the Murdoch University Human Research Ethics Committee (Permit No. 2017/196), and the research team obtained written consents from the egg businesses.

## 5. Conclusions

The results of the present study illustrate the high occurrence of *Salmonella* in the surveyed egg business environment in WA. The predominant serovar (*S.* Typhimurium) identified in this investigation was also reported in several human cases of salmonellosis in recent years in WA. The high level of *Salmonella* recovery from egg business environments in WA highlights the need for effective management practices and biosecurity measures, including cleaning and disinfection of sheds, regular *Salmonella* testing programs, rodent control, vaccination, and provision of uncontaminated feed. Insights from the questionnaire may assist the state authority and local governments to develop industry assistance and regulatory (audit/inspection) practices that may prove useful to egg businesses.

## Figures and Tables

**Table 1:** Occurrence of *Salmonella* in table egg layer farms environment in Western Australia (WA) (7 egg businesses, 26 flocks).

Production System	Egg Business	No. of Birds in Each Farm	No. of +ve Flocks/No. of Sampled Flocks (%)	No. of +ve Samples/No. of Collected Samples (%)	Flock Environment Samples (No. Positive/No. Tested)						
					Pooled Faecal Sample	Boot Swab—Inside	Boot Swab—Outside	Dust	Feed—Inside Shed	Feed—Outside Shed/Silo	Water—Inside Shed
Barn-laid	A	27,000	4/5	8/49 (16.3)	—	2/10	2/4	3/10	1/10	0/5	0/10
	B	32,000	2/2	11/24 (45.8)	3/6	3/4	—	4/4	1/4	0/2	0/4
	C *	40,000	0/2	0/18 (0.0)	—	0/4	—	0/4	0/4	0/2	0/4
	E	12,000	6/6	23/63 (36.5)	15/27	—	2/6	6/12	0/6	0/6	0/6
Overall barn-laid			12/15 (80.0)	42/154 (27.2)	18/33 (54.5)	5/18 (27.7)	4/10 (40.0)	13/30 (43.3)	2/24 (8.3)	0/15 (—)	0/24 (—)
Free-range	C *	40,000	2/2	4/20 (20.0)	—	2/4	1/2	1/4	0/4	0/2	0/4
	D	14,200	1/1	3/11 (27.2)	—	0/2	1/2	2/2	0/2	0/1	0/2
	F	20,000	5/5	29/50 (58.0)	—	5/10	3/5	8/10	8/10	5/5	0/10
	G	15,000	3/3	15/30 (50.0)	—	5/6	0/3	4/6	4/6	2/3	0/6
Overall free-range			11/11 (100.0)	51/111 (45.9)	—	12/22 (54.5)	5/12 (41.6)	15/22 (68.1)	12/24 (50.0)	7/11 (63.6)	0/22 (—)
Totals			23/26 (88.4)	93/265 (35.0)	18/33 (54.5)	17/40 (42.5)	9/22 (40.9)	28/52 (53.8)	14/46 (30.4)	7/26 (26.9)	0/46 (—)

* Business C had both production systems: two barn-laid flocks and two free-range flocks.

**Table 2:** Co-detection of multiple *Salmonella* serovars in table egg layer farm environments in WA (7 egg businesses, 26 flocks).

Production System	Egg Business	No. of +ve Flocks/No. of Sampled Flocks (%)	Serovar Diversity Pattern in Each Visited Egg Business	No. Flocks With the Serovar Pattern
Barn-laid	A	4/5	*S.* Orion	2
			*S.* Tennessee, *S.* Alsterdorf subs. II	1
			*S.* Muenchen	1
	B	2/2	*S.* Infantis	1
			*S.* Infantis, *S.* Kiambu, *S.* Tennessee	1
	C	0/2	*—*	—
	E	6/6	*S.* Typhimurium	6
Free-range	C	2/2	*S.* Typhimurium	1
			*S.* Typhimurium, *S.* Choleraesius v. Decatur	1
	D	1/1	*S.* Typhimurium	1
	F	5/5	*S.* Typhimurium	1
			*S.* Typhimurium, *S.* Infantis	3
			*S.* Typhimurium, *S.* Infantis, *S.* Anatum	1
	G	3/3	*S.* Typhimurium	1
			*S.* Typhimurium, *S.* Infantis	2

**Table 3:** Production and farm management practices of the egg businesses.

Question Categories	Variables	No. (%) of Egg Businesses
Locality	Metro	5 (71.4)
	Out-metro	2 (28.6)
Production system	Barn-laid	3 (42.9)
	Free-range	4 (57.1)
Quality management system		
Does your business have Quality Assurance/Food Safety Management Statement in place?	Yes	7 (100.0)
	No	—
Does your business have a HACCP (Hazard Analysis and Critical Control Points) based food safety program in place?	Yes	7 (100.0)
	No	—
Who is responsible on HACCP management in your business?	External agent/service	1(14.3)
	Onsite dedicated staff	6 (85.7)
Feed and water supply		
How do you source stockfeed used for laying hens?	From manufacture or supplier	7 (100)
	Assemble dry mash or pelleted feed onsite	—
What is the name of the feed supplier?	Supplier A	3 (42.8)
	Supplier B	2 (28.6)
	Supplier C	2 (28.6)
Do you test incoming feed for *Salmonella*?	Yes (sometimes)	1 (14.3)
	No	6 (85.7)
What is the source of water provided for laying hens?	Non-reticulated water, bore water	4 (57.1)
	Non-reticulated water, dam water	1 (14.3)
	Reticulated water supply (scheme water)	2 (28.6)
Layer management		
Are birds single- or multi-aged?	Single-aged	7 (100)
	Multi-aged	—
Do you source replacement birds from single or multiple suppliers?	Single	7 (100)
	Multiple	—
What is the name of the bird supplier?	Supplier X	2 (28.6)
	Supplier Y	4 (57.1)
	Not answered	1 (14.3)
Do you require replacement birds to be vaccinated against *Salmonella*?	Yes	1 (14.3)
	No	4 (57.1)
	Sometimes	1 (14.3)
	Not answered	1 (14.3)
Do you request a declaration from the hen stock supplier that chicks are tested free from *Salmonella*?	Yes	4 (57.1)
	No	2 (28.6)
	Not answered	1 (14.3)
Do you routinely take microbiological samples for testing for *Salmonella* in layers farm environment throughout production age?	Yes	4 (57.1)
	No	3 (42.9)
Do you routinely take microbiological samples for verification testing of cleaning and sanitisation program between flocks?	Yes	1 (14.3)
	No	5 (71.4)
	Not answered	1 (14.3)
Aspects of biosecurity management		
Does your business have a policy of restricted person’s access (staff and verified visitors only)?	Yes	7 (100.0)
	No	—
For visitors, do you have a policy of insisting on a 48- to 72-hour delay in between farm visits?	Yes	4 (57.1)
	No	3 (42.9)
For visitors, do you maintain vehicle wheel wash?	Yes	1 (14.3)
	No	6 (85.7)
At each shed entry, do you maintain footbaths filled with sanitiser?	Yes	5 (71.4)
	No	2 (28.6)
At each shed entry, do you maintain handwashing facilities (basin)?	Yes	5 (71.4)
	No	2 (28.6)
Before shed entry is there an ante-room facility?	Yes	5 (71.4)
	No	2 (28.6)
Are workers and visitors required to wear personal protective equipment (PPE) and/or specific protective clothes before entering the shed?	Yes	3 (42.9)
	No	4 (57.1)
Are workers and visitors required to change their clothes while walking between sheds?	Yes	1 (14.3)
	No	5 (71.4)
	Not answered	1 (14.3)
Do wild animals and birds have access to laying sheds?	Yes	3 (42.9)
	No	4 (57.1)
Do rodents have access to laying sheds?	Yes	6 (85.7)
	No	1 (14.3)
Do domestic pets have access to laying sheds?	Yes	—
	No	7 (100.0)
Do you keep other livestock on the farm?	Yes	3 (42.9)
	No	4 (57.1)
Egg handling practices and processes		
What is the egg collection method used by business?	Fully automated	1 (14.3)
	Semi-automated	3 (42.8)
	By hand	3 (42.8)
What is the egg grading method used by business?	Fully automated	1 (14.3)
	Semi-automated	3 (42.9)
	By hand	3 (42.8)
How frequently are eggs collected on a daily basis?	Once/day	3 (42.9)
	Twice/day	1 (14.3)
	Three times and more/day	3 (42.8)
How long is the storage period for collected eggs before egg grading (time between collections to grading)?	Within 24 hrs	4 (57.1)
	Up to 48 hrs	3 (42.9)
What is the typical storage temperature of pre-graded eggs?	Ambient	4 (57.1)
	≤15°C	3 (42.9)
How often do you sanitise egg handling equipment?	Daily	4 (57.1)
	Weekly	1 (14.3)
	Monthly	1 (14.3)
	Not answered	1 (14.3)
Which of the following best describes the hairline crack detection system you have in place?	Visual	3 (42.9)
	Candling	4 (57.1)

**Table 4:** Methodology for environmental sample collection from table egg layer farms.

Sample Type	Collection Methodology from Each Flock
Pooled faecal material	Approximately 200–300 g or 40 fecal pinches were collected from different areas of the shed floor or deep pit.
Dust	Approximately 50 g of dust was collected from 40 different surfaces with a visible dust presence, including ledges, tops of nest boxes, and ventilators, inside each shed and placed into Whirl-Pak sample bags.
Boot swab	Boot swabs were worn over sterile plastic boot covers and sprayed with sterile water. Approximately 100 shuffling steps were taken over different parts of bird access areas inside and outside of sheds.
Feed	Approximately 250 g of feed was collected from feed troughs and feeder silos.
